# Endoscopic Management of an Internal Laryngopyocele Presenting with Acute Airway Obstruction

**DOI:** 10.1155/2011/873613

**Published:** 2011-08-04

**Authors:** Zenon Andreou, Premjit S. Randhawa, Paul O'Flynn, Francis M. Vaz

**Affiliations:** Department of Head and Neck Surgery, University College London Hospital NHS Trust, London NW1 2PG, UK

## Abstract

*Statement of Problem*. Laryngocele is a rare laryngeal disease, where there is an abnormal dilatation of the saccule of the laryngeal ventricle. It can either be internal or external, and a laryngopyocele is a rare complication of this anomaly. Internal laryngopyoceles can prove difficult to manage, as they often present with airway compromise. *Method of Study*. Case Report. *Results*. We present a case of a laryngopyocele that was successfully managed with suspension laryngoscopy and endoscopic marsupialisation and resection. To our knowledge, this is the first such case described in the literature. *Conclusions*. Surgical drainage of a laryngopyocele via the external approach is well documented in the literature. We feel that endoscopic resection of laryngopyoceles in an emergency situation is a viable alternative and also prevents the associated surgical morbidity.

## 1. Introduction

Laryngocele is a rare laryngeal disease, where there is an abnormal dilatation of the saccule of the laryngeal ventricle [1]. It can be external, where the space expands laterally and breaches the thyrohyoid membrane presenting as a neck mass, internal, where it expands medially entering the supraglottic space, or mixed, where there is a combination of the two presentations. When it occurs, it is a potential space in the neck that has the risk of getting infected and filling with mucopus thus becoming a laryngopyocele [2].

We report a case of an internal laryngocele, probably present and quiescent for some time, that presented as an emergency with acute airway compromise as a laryngopyocoele. The patient was treated successfully with initial intubation and subsequent endoscopic incision, drainage, and marsupialisation of the cyst.

## 2. Case Report

A 63-year-old previously fit and healthy woman, presented to the Accident and Emergency Department with acute onset stridor and difficulty breathing. She had been feeling unwell for the previous two days and had put her symptoms down to a common cold. She had a background of hoarse voice for more than a year for which she never sought medical attention. On arrival to the department she was found to be stridulous and in respiratory distress. She received immediate airway management in the A&E with one millilitre of Adrenaline mixed with four millilitres of normal saline nebulised and 200 milligrams of Hydrocortisone intravenously. On examining her larynx with a flexible nasal endoscope, a limited view of the glottis was obtained secondary to significant supraglottic oedema. There were no signs of systemic sepsis although her initial haematological and biochemical investigations revealed nonspecific inflammation with elevated levels of C-reactive protein (25.6 mg/L) and erythrocyte sedimentation rate (48 mm/h). She did not respond to conservative treatment and was, thus, intubated with a size 6.0 Fr endotracheal tube with the help of the ENT team. 

Direct laryngoscopy at the time of intubation revealed a mass over her right glottic area which was almost completely obscuring the laryngeal inlet. She was then transferred to the intensive care unit, where she remained sedated and ventilated. She was treated with intravenous antibiotics for a suspected supraglottitis given her clinical picture and the appearance of her larynx. 

Over the next 48 hours, it became apparent that there was no demonstrable leak around her endotracheal tube cuff despite medical management. A decision was made to perform a microlaryngoscopy and to attempt extubation in theatre. 

**Figure 1:**
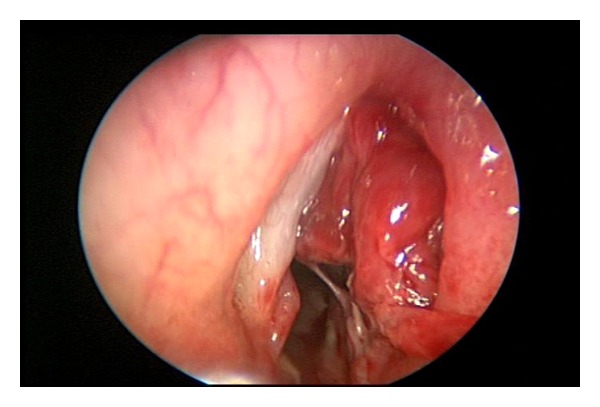
Endoscopic view of the right laryngopyocele before marsupialisation and excision.

Suspension laryngoscopy was performed with supraglottic jet ventilation, and a bulging mass was then seen protruding over her right subglottic area and partially obstructing her laryngeal inlet (Figure 1). She was reintubated, and her airway secured with a surgical tracheostomy. Her larynx was then revisited, and after careful examination, a diagnosis of a laryngopyocoele was made. The laryngopyocoele was de-roofed using long laryngeal biopsy forceps, and 10 millilitres of frank pus was immediately aspirated from the area. The excess mucosa comprising the remaining laryngocele was excised and sent to histology. Histological examination of the specimen revealed necrotic and infracted tissue with a very slight possibility of malignancy. The pus aspirated from the cavity grew *Staphylococcus aureus* sensitive to Penicillin and Flucloxacillin. The patient then returned to ITU and made a very swift recovery on intravenous antibiotics, leaving ITU the next day. 

**Figure 2:**
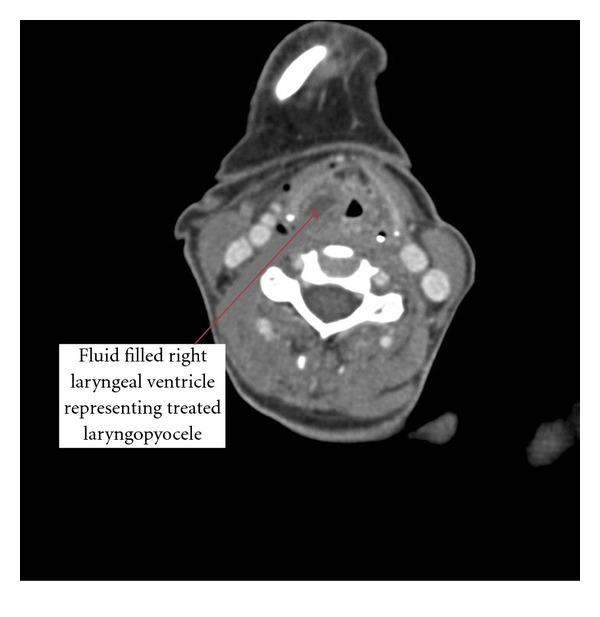
CT image immediately postoperatively with evidence of the marsupialised laryngopyocele on the right.

A CT scan was performed following the procedure revealed that the mass was adequately drained, and no external component was identified (Figure 2). 

**Figure 3:**
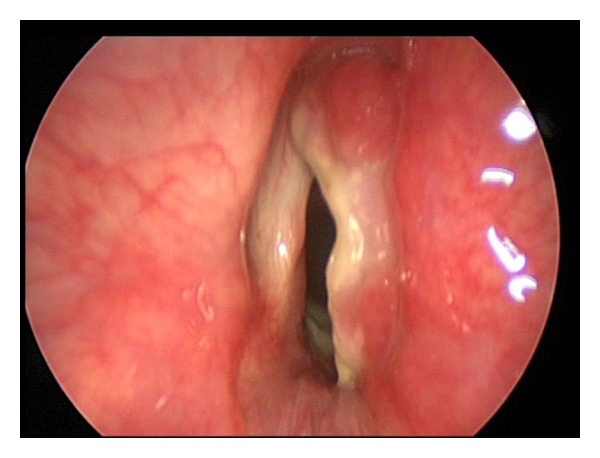
Endoscopic view of larynx 14 days after treatment with evidence of good healing.

The patient underwent a repeat microlaryngoscopy and biopsy of the area 14 days later, which showed good healing (Figure 3). Further histological analysis revealed no evidence of malignancy. She was, therefore, decannulated and discharged home shortly after that. On reviewing her larynx in clinic four weeks later, the inflammation appeared to be resolving with only a small amount of granulation tissue in the supraglottis. There was also significant subjective improvement in the voice quality.

## 3. Discussion

A laryngocele is an abnormal dilatation of the laryngeal saccule, first described in the early 19th century by Virchow. Following that, there have been multiple accounts of laryngoceles in the literature although the condition remains fairly rare. It can be asymptomatic so its true incidence is very difficult to calculate. It is estimated that about 2.5 per million symptomatic laryngoceles occur per annum in the United Kingdom [3]. Most commonly, it is a congenital abnormality, but it has been demonstrated to arise in people with prolonged periods of increased laryngeal pressure such as glass blowers and wind instrument players [4, 5]. Laryngoceles can be internal, external, or mixed. An association of laryngoceles with squamous cell carcinoma of the larynx has been well established, necessitating a high index of suspicion for malignancy when present [6]. 

Symptoms of laryngoceles depend on the subtype. External laryngoceles usually present as a neck mass, which varies in size according to how much air is in the saccule at any one time. Internal laryngoceles present with laryngeal symptoms such as hoarse voice, foreign body sensation, and sore throat. Very rarely, a laryngocele can become infected and turn into a laryngopyocoele with only around 50 cases so far reported in the literature [7, 8]. The air-filled pouch can get blocked by mucus and continue to enlarge. When that mucus collection gets infected, a laryngopyocele forms. In cases of external laryngoceles, an external laryngopyocele will present with an infected neck mass and will be managed accordingly. 

Internal laryngopyoceles are exceedingly rare, with only a handful of cases reported in the literature [9]. They usually prove very challenging to manage. They often present in extremis, as in the case of the patient presented above, with a very unstable airway due to the nature of the disease. A review of the current literature reveals that there is no consensus on how to manage such patients, due to the rarity of the presentation. As it is evident, securing an airway is paramount in every case, and in most cases in the literature, the patient is managed with an urgent tracheostomy and resection of the laryngocele via an external approach [2, 10]. There have been cases in the literature where a laryngopyocoele has been managed with initial ultrasound-guided aspiration of the pus-filled cyst to relieve the acute symptoms with formal excision of the laryngocele at a later stage [11]. However, despite the fact that an endoscopic approach to noninfected internal laryngoceles has been established with very good results, there have not been any reports of laryngopyoceles being managed endoscopically [12, 13].

In our case, we managed the laryngopyocele via an endoscopic approach after securing the airway with a tracheostomy. This provided an effective way of making a diagnosis in an otherwise complex situation and secondly managing the problem definitively by marsupialising, draining, and excising the laryngopyocoele during the same anaesthetic episode. Notably, the patient did not experience any complications; she did not develop any pneumonia/pneumonitis, which is always a worry when dealing with intralaryngeal abscesses. She was weaned of the ventilator within 24 hours and stopped antibiotic therapy within 5 days, as she made very good recovery. She was successfully decannulated two weeks after the procedure after a repeat biopsy of the area did not reveal any malignancy. 

We advocate the approach of endoscopic resection of laryngopyoceles in emergency situations, as it can expedite the patients' treatment by effectively diagnosing and managing such conditions. Moreover, endoscopic resection offers an alternative to the old approach of external resection of laryngoceles and prevents the associated surgical morbidity.
